# Contemporary empyema thoracis necessitans in an adult male caused by *Staphylococcus aureus*: decortication is superior to traditional under water seal intercostal tube in chronic empyema

**DOI:** 10.11604/pamj.2015.20.115.5865

**Published:** 2015-02-09

**Authors:** Liaqat Ali Chaudhry, Ahmed A Ba Mousa, Marwan Zamzami, Asirvatham Alwin Robert

**Affiliations:** 1 Department of Internal Medicine, Pulmonary Division, Sultan Bin Abdulaziz Humanitarian City, Riyadh, Saudi Arabia; 2 Department of Surgery, Prince Sultan Military Medical City, Riyadh, Saudi Arabia; 3 Department of Orthopedic Surgery, College of Medicine, King Saud University, Riyadh, Saudi Arabia; 4 Department of Endocrinology and Diabetes, Diabetes Treatment Center, Prince Sultan Military Medical City, Riyadh, Saudi Arabia

**Keywords:** Empyema, paraplegis, Staphylococcus aureus

## Abstract

Empyema thoracis necessitans is a rare clinical finding nowadays. We report 55 years old Saudi male with past history of road traffic accident, poly trauma, chest surgery and paraplegia admitted for rehabilitation in Sultan Bin Abduaziz Humanitarian City (SBAHC), Riyadh, Saudi Arabia and diagnosed with empyema thoracis necessitans due to Staphylococcus aureus, treated initially with traditional thoracostomy under water seal intercostal intubation and antibiotics but subsequently required decortication.

## Introduction

Thoracic empyema is a disease of substantial morbidity and mortality, mainly in the developing world where tuberculosis remains a common cause [1]. It is known to be associated with prolonged respiratory symptoms requiring longer times for drainage due to excessive fibrosis and pleural thickening [2]. Suppurative bacterial infections are also an important cause of empyema, which if not diagnosed and treated early by antibiotics alone or with early under water seal intercostal tube, may result in to chronic empyema or even empyema thoracis necessitans requiring open drainage and decortication. Empyema necessitans is spillover of pleural space infection or pus in to soft tissue of the thoracic wall. It may burst out via weak part of the chest wall to skin and start discharging pus; otherwise it may involve surrounding structures in any direction. After *Streptococcus pneumonia, Staphylococcus aureus*, is the commonest cause of empyema in children, but it is not uncommon in adults either [3]. Chronic suppurative empyema is more common and even recurrent in patients with past history of poly trauma or chest surgery. Chronic empyema treated with decortications results in rapid resolution of infection and better re-expansion of the lung as compared to only under water seal intercostal tube [4]. We report 55 years old Saudi male with history of road traffic accident, poly trauma, chest surgery and paraplegia admitted for rehabilitation in Sultan Bin Abduaziz Humanitarian City (SBAHC), Riyadh, Saudi Arabia and diagnosed with empyema thoracis necessitans due to *Staphylococcus aureus*, treated initially with under water seal intercostal intubation and antibiotics but subsequently required decortication.

## Patient and observation

**Figure 1:**
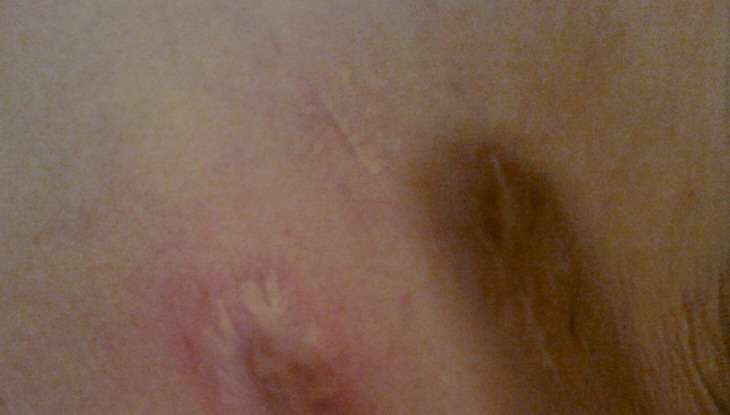
Old thoracostomy scars

**Figure 2:**
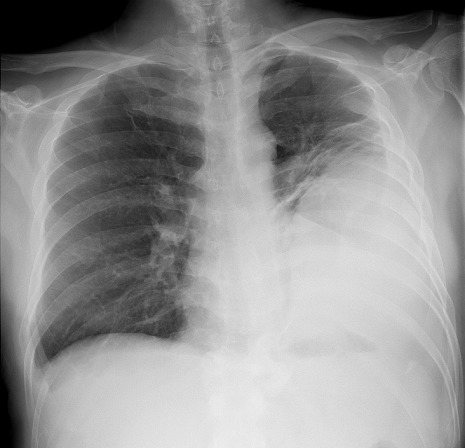
Chest X ray on admission with left sided thick biconvex opacity

**Figure 3:**
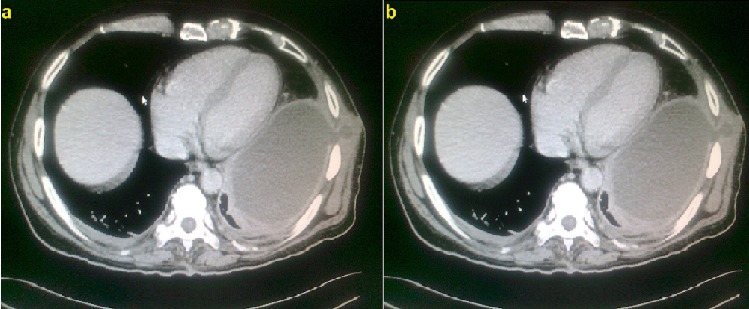
(a and b) CT scan chest showing left sided chronic empyema

**Figure 4:**
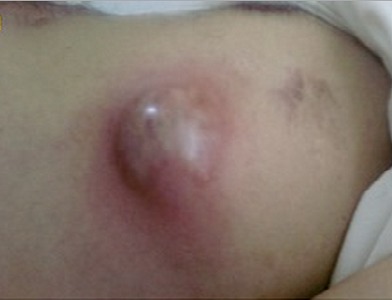
Umblicated yellow red pre-rupture stage empyema thoracis necessitans

A Saudi male 55 years of age suffered poly trauma in a road traffic accident including chest two years ago and was admitted in SBAHC for rehabilitation for having paraplegia on 16-6-2013. Three days since admission he was running fever and his chest x-ray on admission being abnormal was referred a bit late for pulmonary consultation on 19-6-2013, he was being treated by the primary team as pneumonia. He had mild cough, feeling of heaviness and discomfort on the left lateral chest without significant expectoration. He had poor oral hygiene, malodorous fetor with pyorrhea and dark brown deposits on the teeth. On systemic examination his BP was 120/70, HR = 88, RR = 19, T = 38.5, O^2^ Saturation = 95% at room air, Weight = 78 Kg. There were obvious old thoracostomy scars (Figure 1) performed two years ago on the left chest wall. Percussion note was dull and reduced breath sounds on the ipsilateral side. Percussion note was stony dull. There was no paradoxical, respirartion and heart rate pattern on clinical examination as well as on electrocardiogram. Echo cardiac examination was unremarkable with no out flow obstruction apart from mild left ventricular diastolic dysfunction consistant with his age. His chest x-ray (Figure 2) showed left sided thick biconvex opacity occupying left lower and middle zone. Lab works showed Hb = 12gm, RBC = 3.6, WBC = 12.6, N = 88, L = 10, M = 1, E = 1, PLT = 366, ESR = 32, BU = 6.2, SCR = 76, LDH = 40, CRP = 16, PPD = 5mm. Under the provisional diagnosis of chronic empyema patient was started on empirical injection clindamycin 600 mg iv q8hrs. Culture on blood, urine and sputum was awaited. After having CT scan chest (Figure 3) an urgent external thoracic surgical consultation was requested as our hospital is a rehabilitation facility there is no thoracic surgical specialty. The color of an old thoracostomy scar started changing dark brown to red color and in next 24-48 hours it became umblicated and yellow (Figure 4). On the 7^th^ day of admission on 23-6-2013 while he was being examined by the thoracic surgeon he had an intractable bout of cough which lead to spontaneous rupture with pouring out of frank pus. A specimen of the pus was sent for bacterial and mycobacterial culture besides Acid Fast Bacteria (AFB) direct smear microscopy. Patient felt relieved and had no adverse events like breathlessness or pain. He was taken to the operation room and was inserted underwater intercostal tube as part of traditional thoracostomy.

**Figure 5:**
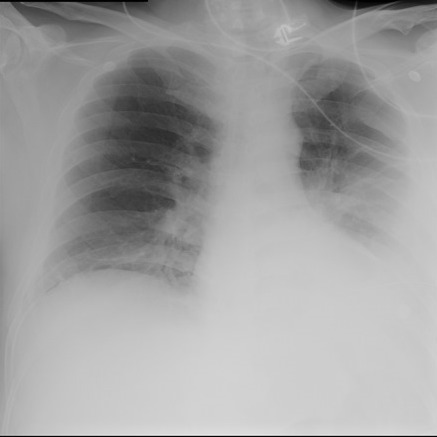
Post ICT Chest X ray showing non expanded left lung

**Figure 6:**
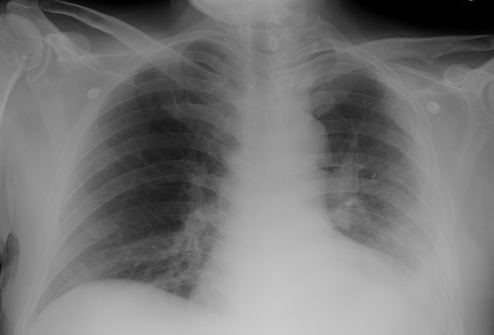
Post decortication chest X ray showing complete re expansion

Patient was sent back to the unit where he felt better his temperature became less but his lung remained unexpanded with residual loculations and still residual pus on post intercostal tube (ICT) chest x-ray (Figure 5). Culture on the pus, was reported heavy growth of *Staphylococcus aureus*, sensitive to clindamycin so same antibiotic was continued. Pus was reported AFB negative on direct smear microscopy. Blood and urine culture were reported negative. Next day the patient was taken for decortication and was kept subsequently in the high dependency unit (HDU). He remained stable initially on ventilator for 48 hours, then on spontaneous breathing and oxygen subsequently maintaining normal range O^2^ saturation at room air. His lung remained well anchored and expanded with no complications, hence all three one anterior apical, and two posterior apical and basal under water seal drainage tubes along with Robert's pump application were removed one by one. Culture on pus for Mycobacteria was reported negative. Patient remained stable made good uneventful recovery and was discharged home on 16-7-2013, his last chest x-ray shown (Figure 6). There was complete re-expansion of the lung with no residual collection. He was advised OPD follow up which he failed.

## Discussion

Respiratory infections are a common cause of pleural effusion. Overwhelming chest infections, virulent strains and delay in diagnosis and antibiotics treatment result in complications [5]. The assessment of those having parapnemonic pleural effusion or empyema require careful history, physical examination, radiological imaging (CXR, Ultrasound, CT Chest) and lab works (microbiology, cytology, biochemistry) [6]. Empyema may take origin from parapneumonic pleural effusion (stage-I) when fluid may still be sterile with low leukocyte count (<50%), low LDH levels, having a PH in normal physiological range and usually normal glucose levels. If not treated early with appropriate antibiotics it may progress to stage-II (fibropurulent) with high leukocytes, high LDH, low PH (<7) and low glucose. If still not treated with antibiotics or fibrinolytics and intercostal drainage, it may further enter in to organizational stage-III with extensive fibrositic activity and fibrin deposition with loculation and septation. Pus formation under pressure may track its way to adjacent structures or to the chest wall as seen in this in this case [6]. This usually results in restrictive ventilatory dysfunction (trapped lung) and usually requires decortication with better outcomes as in our patient. Pneumonias are more common at extremes of age. Streptococcal pneumonia and mycobacterium tuberculosis are the commonest organisms reported in community acquired *Pneumonias,while klebsiella pneumoniae, Staphylococcus aureus,, MRSA and Pseudomonas* are the organisms encountered in nosocomial pneumonias. Viral respiratory infections are common in children and are often complicated with bacterial pneumonias, pl.effusion, empyema and *Staphylococcus aureus*, being the commonest organism. In adults having pl.effusion or empyema, organisms differ from those having community acquired pneumonia. In a study 68% adults reported positive cultures for streptococcus milleri 19%, bacteroides 14%, klebsiella pneumonia 12% and peptostreptococcus 7% respectively [7]. In adults having past history of chest trauma or thoracic surgery *Staphylococcus aureus* is the commonest organism encountered as in the subject patient [8].

Patients having chronic empyema usually has indolent course and has none to few symptoms resulting in delayed diagnosis as in this patient. Conservative medical treatment with antibiotics is not adequate and early thoracic surgical intervention is warranted. In the absence of early surgical intervention there is always a risk of rupture of empyema in to surroundings or outside via chest wall (empyema thoracis necessitans) more so in those with past history of thoracic surgery as occurred in this patient. Whereas stage I and II empyemas are amenable by antibiotics and fibrinolytics or under water seal intercostal drainage, an advanced stage- III organized empyema requires decortication resulting in improved lung expansion, less chances of recurrence and better outcomes as in this case [9, 10]. The patient was being treated by the primary team as left sided pneumonia and was referred a bit late by the primary team as non-resolving pneumonia for pulmonary consultation having abnormal chest x-ray (Figure 2) and running fever since admission, with negligible cough, no expectoration, but having a sense of heaviness on the left chest wall. The patient had past history of poly trauma in a road traffic accident including hemopnemothorax on the left side and a history of thoracic surgery and thoracostomy tube on the left side 24 months ago leaving scars (Figure 1). He sustained spinal cord injury having fractured T12 vertebrae resulting in to paraplegia and thus was admitted to SBAHC, Riyadh for rehabilitation. He had very bad mouth hygiene which could be another contributing factor.

After reviewing his history, clinical chest examination and chest x-rays under the provisional diagnosis of chronic empyema he was started empirical treatment with injection. clindamycin 600 mg iv q8h and an urgent thoracic consultation was arranged. The old scar on the left chest wall started showing changes not only in its color but started swelling and became umbilicated (Figure 4). Our facility is a rehabilitation hospital, it took about 4 days for the thoracic surgeon to attend the patient from outside, during examination by the thoracic surgeon patient had a bout of intractable cough which resulted in the spontaneous rupture of the empyema thoracis necessitans. A sample of this pus was sent for direct smear AFB, pyogenic bacterial and Mycobacterial culture. The patient was not in any distress, rather he felt relieved of pre-eruptive heaviness felt on the left chest wall. He was immediately taken to the operation theatre and after traditional thoracostomy under water seal intercostal tube was inserted and was sent back to the unit. Post intercostal tube insertion chest x-rays was showing persisting thick septations and trapped left lung. After another 30 hours on 24-6-2013 patient was re-operated for decortication and was kept in HDU on mechanical ventilation. A formal left postolateral thoracostomy sparing serratus anterior muscle was preferred over VATS, as VATS will not be optimal treatment in this condition at this stage of chronic empyema. The underlying lung was healthy having no lesions. Three intercostal drainage tubes apical left posterior, 2^nd^ anterior apical,3^rd^ lower basal midaxillary region were inserted with Robert pump application. Three intercostal drainage tubes were used to facilitate complete drainage of pus. The pus culture reported positive for *Staphylococcus aureus*, heavy growth sensitive to clindamycin which was continued. Pus was negative for AFB on direct smear microscopy and Mycobacterial culture, Patient became afebrile on 8th day of antibiotics especially after drainage of the empyema.

Post decortication chest x-rays showed complete re-expanded and well anchored left lung with no residual collection (Figure 6). After 72 hours patient was gradually weaned off the ventilator. His progress remained uneventful. His drainage in the chest tubes gradually decreased. On 4^th^ day day there being no discharge through the top intercostal tube, it was removed, and after another 3 days all intercostal tubes were removed. Patient showed very good recovery and having normal oxygen saturation at room air with no respiratory complaints was discharged home in stable condition after staying 23 days in the HDU. Oral antibiotics were continued for another 4 weeks. Patient was advised OPD follow up after 4 weeks. He failed to follow, his residence being far away from Riyadh area.

## Conclusion

*Staphylococcus aureus* although more common in children, remains a common pathogen in adults who are alcoholics, or has past history of chest trauma and previous thoracic surgery as in this patient. Whereas all empyemas require early recognition and prompt treatment with appropriate antibiotics, advanced chronic empyemas (stage III) in addition require surgical intervention, where decortication is superior to traditional under water seal intercostal drainage, allowing better outcomes reflected by better lung re-expansion and hence improved lung function.
